# Artificial Alimentation

**Published:** 1887-03

**Authors:** 


ARTIFICIAL ALIMENTATION.—Dr. G. D. Hays concludes an article in the *N. Y. Medical Journal*, January 27, 1887, on this subject as follows:

1. Many pathological processes arise from an improper dietary, and many others may be controlled by a proper dietary. There is a celebrated proposition by M. Broussais: "He who does not know how to manage the stomach will never know how to treat diseases."

2. Malassimilation is a cause of disease. Peptones in the general circulation being poisons, when from any cause metabolism is interrupted, the system is prone to take on pathological conditions.

3. In pyrexia: *a*. The digestive juices, being less in quantity and impaired in quality, should be re-enforced by the artificial digestive ferments.

*b*. The stomach is feeble in a muscular sense, and incapable ot dealing with large quantities, hence, concentrated or predigested foods should be furnished it.

*c*. The process is one of tissue destruction (histolytic), hence, we must furnish the materials for repair (histolysis). How and when to furnish these is still a disputed question.

*d*. Feeding is not the cause of pyrexia, nor starvation its cure. Though the former augments it and the latter decreases it, we gain most by keeping up nutrition.

*e*. The excess of urea excreted is not proof of nitrogenous waste in the blood.

